# Turn-taking in grooming interactions of chimpanzees (*Pan troglodytes schweinfurthii*) in the wild: the role of demographic and social factors

**DOI:** 10.1007/s10071-025-01940-7

**Published:** 2025-03-24

**Authors:** Kayla Kolff, Simone Pika

**Affiliations:** Comparative BioCognition, Institute of Cognitive Science, Osnabrück University, Osnabrück, Germany

**Keywords:** Language evolution, Coordination, Grooming, Chimpanzees, Social accommodation, Age, Relatedness, Dominance rank, Social bonds

## Abstract

Cooperative turn-taking, a fundamental characteristic of human social interaction, has been postulated as a crucial mechanism for language emergence and is observed across the primate lineage. However, relatively little is known about the influence of demographic and social factors on turn-taking. As according to the sociolinguistic *Communication Accommodation Theory*, individuals adapt their communication according to their recipient characteristics, which may shape turn-taking. Thus, we aimed to gain insights into the factors (age, relatedness, dominance rank, and social bonds) in relation to the turn-taking infrastructure of one of our closest living relatives, the chimpanzee (*Pan troglodytes schweinfurthii*) of the Ngogo population in Kibale National Park, Uganda. We specifically focused on a cooperative context, grooming, including gestures and actions, and collected data over nine months (September 2021 to June 2022) involving 42 male chimpanzees. We analysed 311 grooming interactions among 157 dyads concerning the role of demographic and social factors in turn transition infrastructure, turn transition types, and temporal relationships. Our findings demonstrated that turn transitions and types were influenced by age and dominance rank, whereas social bonds and relatedness did not exhibit effects. Specifically, the probability of turn transitions was higher for older initiators and lower-ranking or younger recipients. These effects varied across turn transition types, where initiator’s dominance rank and relatedness showed no effects on any type. In addition, no effect was found for the temporal relationships. Although the social dynamics of turn-taking remain largely unexplored across both human and non-human studies, our findings suggest that turn-taking can occur selectively between certain individuals, in line with the *Communication Accommodation Theory*, underscoring the need for greater focus on investigating how demographic and social factors shape turn-taking.

**Supplementary Information:**

The online version contains supplementary material available at 10.1007/s10071-025-01940-7.

## Introduction

Language, a uniquely human trait and cooperative layered system, has been a topic of interest across centuries and research disciplines because its emergence remains a scientific puzzle (Christiansen and Kirby 2003; Fitch 2010; Hauser et al. 2014; Knight et al. 2000). Recently, it has been proposed that the special capacity for social interaction among humans facilitated the evolution of language (Levinson 2006, 2016; see also Vygotsky 1962). This capacity rests on a layered assemblage of different social cognitive skills (e.g., joint attention, common ground, collaboration, and reasoning about communicative intent; Clark 1996), and involves face-to-face interaction, frequent employment of mutual gaze, and turn-taking, forming the “interaction engine” (Levinson 2006). In particular, turn-taking, a cooperative enterprise (Sacks et al. 1974), has recently received much research attention because it has been suggested as an ancient underpinning of the language system, with precursors already present across the primate lineage (Levinson 2016). Turn-taking refers to reciprocal exchanges of alternating short and flexible turns between two or more interactants and is governed by distinct features: one interactant speaks at a time, the organisation of allocated turns between interactants are varied, temporal gaps are minimised (approximately 200 ms; Stivers et al. 2009), and overlaps are avoided (Sacks et al. 1974).

Turn-taking is suggested to be a universal enterprise found across human languages and cultures (Stivers et al. 2009); however, the social identities of the interactants may also influence turn-taking, in line with the sociolinguistic *Communication Accommodation Theory* (Giles 2008; Giles and Powesland 1975). This theory posits that individuals adapt their communication styles, to align or “accommodate” with their intended recipient based on factors like social bonds (e.g., Farley et al. 2013). For instance, mothers have been shown to shape turn-taking interactions with their infants (e.g., Gratier et al. 2015). Therefore, in accordance with *Communication Accommodation Theory*, turn-taking may be influenced by and adapted to the social context and relationships between interactants and may extend beyond human communication.

Some scholars have proposed that turn-taking may provide an evolutionary “missing link” in communication between non-human species and humans (Levinson 2016), where a more sophisticated purely signal infrastructure represents turn-taking in human conversations (see Rossano 2018). Recent comparative studies have shown that some forms of turn-taking are also present in non-human animals, primarily primates, on their exchanges, demonstrating overlap avoidance and sequence organisation (e.g., Fröhlich et al. 2016; Martin et al. 2017; Rossano 2013; Snowdon and Cleveland 1984; Takahashi et al. 2013). Hence, some studies have investigated the influence of demographic and social factors on turn-taking. For example, a study on captive Campbell monkeys (*Cercopihecus campbelli campbelli*) suggested that some elements of turn-taking might be linked to development and learning, with only adult individuals adhering to overlap avoidance (Lemasson et al. 2011). A study on wild white-faced capuchins (*Cebus Capucinus*) revealed that irrespective of sex, dominant individuals were more likely to elicit a vocal response from their conspecifics (Digweed et al. 2007). Concerning social bonds, a study on zoo-housed bonobos (*Pan paniscus*) showed that individuals responded preferentially to the call of a conspecific with whom they shared a strong social bond (Levrero et al. 2019). However, these studies primarily focused on purely vocal exchanges.

There is currently a lack of understanding about the role of demographic and social factors in other turn-taking modalities, such as gestures and actions (referring to any socially directed behaviours that lead to the intended goal through the direct manipulation of another’s body or the movement of one’s own body; Fröhlich, Wittig, et al., 2016). However, this is essential for assesing the complexity involved, the multimodal behavioural repertoire of a social interaction (Mondada 2016), and their similarities and differences to human conversational turn-taking.

Hence, the present study aimed to address this gap by investigating the impact of demographic and social factors on the infrastructure of nonvocal turn-taking. For this purpose, we focused on the grooming context of one of our closest living relatives, chimpanzees (*Pan troglodytes*). Grooming, suggested to serve a social bonding function that may have paved the way for language (Dunbar 1996, 2004), provides a cooperative context and includes a tit-for-tat organisation (i.e., role reversal: Machanda et al. 2014; Mitani 2006), negotiations, and a complex array of gestures (Pika 2014). It permeates nearly every aspect of chimpanzee societies (Goodall 1986), with grooming interactions, especially of male chimpanzees, being governed by demographic and social factors such as age (e.g., younger directed to older: Rosati et al. 2020; Sandel et al. 2020), dominance rank (e.g., lower directed to higher especially in steep-dominant societies: Newton-Fisher and Lee 2011), relatedness (e.g., directed between maternal brothers: Sandel et al. 2020), and social bonds (e.g., directed to bonded partners: Mitani 2009).

Therefore, this present study focused on male eastern chimpanzees (*P. t. schweinfurthii*) and their grooming interactions from the Ngogo population in Kibale National Park, Uganda. Based on the evidence of the influence of demographic and social factors on vocal turn-taking of non-human species and general engagement in grooming behaviour, we predicted that these factors may also influence the turn-taking infrastructure of grooming interactions in chimpanzees. In particular, we anticipated an increased likelihood of turn transitions (i.e., role alternation within a grooming interaction) when initiated by higher-ranking individuals, and adults (prime individuals with higher social standing), as they might be more inclined to receive responses, especially from younger individuals or those with a lower rank. In addition, we predicted an increased likelihood of turn transitions between strongly bonded dyads and related dyads. Furthermore, although dominance rank, social bonds, and relatedness may affect temporal relationship, we specifically predicted that adult recipients would show a preference for avoiding temporal overlap with turns because they are socially and interactively more experienced than younger chimpanzees. These predictions are embedded in the *Communication Accommodation Theory* and highlight how communication is dynamic and responsive, shaped by contextual factors and relationships among the chimpanzees involved.

To provide context for our research questions, we first examined turn transitions involving gestures and actions to gain insight into their frequency, types, and temporal relationships of turn transitions within chimpanzee grooming interactions. The turn transitions were classified into four types: action – action (e.g., A grooms B, B grooms A), action – signal (e.g., A grooms B, B produces a gesture), signal – action (e.g., A produces a request gesture, B grooms), and signal – signal (e.g., A produces a gesture, B produces a gesture, a possible form of social negotiation). Thus, the following questions were addressed in this study: What is the role of age, dominance rank, social bonds, and relatedness in the (i) likelihood of turn transitions, (ii) frequency of turn transitions, (iii) types of turn transitions, and (iv) temporal relationships of turn transitions?

## Methods

### Study site and subjects

Data were collected from male eastern chimpanzees from two sub-communities (central and western) of the Ngogo population living in Kibale National Park, Uganda. The area ranges from dry-ground and early- to mid-stage colonising forests to swamp forests and anthropogenic grasslands (Lwanga 2003). The Ngogo chimpanzee population is a profoundly large community of ~ 200 individuals at the time of the study, consisting of ~ 116 individuals in the central community and ~ 84 individuals in the western community at the time of this study.

We observed grooming interactions in 42 males (N_central_ = 27, N_western_ =15) ranging in age from 13 to 56 years. Based on the physical and social attributes of the development of male chimpanzees (Goodall 1986; Kawanaka 1989), the age of 13 was selected to include adolescents in the study (Goodall 1986; Pusey 1990; Reddy and Mitani 2020). This was to incorporate age as a factor in this study, which included individuals who were independent of their mothers. Genealogies have previously been constructed from genetic data at Ngogo (Langergraber et al. 2007), whereby individuals were classified as ‘unrelated’ if they were not maternal siblings.

### Data collection

Data were collected from September 2021 to June 2022, resulting in 160 observation days. We used a focal sampling approach (Altmann 1974) using the Cybertracker software (Version 3.51), while maintaining a record of the total duration for which a focal was observed. In cases where multiple focal individuals were present, priority was given to those who had been sampled less frequently (i.e., lower observation time). During the focal samples, we also collected scan data every 15 min, noting which individuals were within the arm length (approximately one meter) of the focal. In addition, we used ad libitum sampling (Altmann 1974) to collect data on all occurrences of grooming and aggressive interactions, excluding the focal animal. The observational hours per focal male ranged from ten to 41 h (mean = 25.05 ± 10.95 h), resulting in a total of 1064 h (central community = 698.38 h, western community = 365.81 h).

Grooming interactions during the focal follow were recorded using a digital high-definition camera (Sony FDR-AX53 4 K) equipped with an external directional microphone (Sony ECM-GZ1M). Ad libitum recordings of grooming interactions, excluding the focal, were additionally obtained when the focal was resting. We selected a total of 311 grooming interactions (31.33 h), 190 interactions for the central community (20.01 h, mean/dyad = 1.76 ± 1.13), and 121 interactions for the western community (11.31 h, mean/dyad = 2.47 ± 1.88), based on the good visibility of the interactions while accounting for a non-skewed distribution of different dyads to address the research questions. Grooming interactions were divided into three phases: starting, grooming, and ending. The starting phase refers to the initiation of a grooming interaction by one or both of the interactants through the production of a signal (e.g., present^1^) or an action (e.g., approach), with filming starting 30 s before the grooming session. However, not all starting/initiation/solicitation phases could be captured, because not every grooming interaction could be anticipated, and the interaction had already started when the observer arrived at the location. A grooming phase (also referred to as a grooming ‘session’) was defined as a continuous period during which an individual brushes and manually picks through the hair of another interactant with their fingers or mouth (Goodall 1986), without a change in behaviour (e.g., moving, feeding, or resting without grooming; Newton-Fisher and Lee 2011). A grooming phase could entail the switching of grooming roles (also referred to as “bouts”), where a grooming role is defined as an episode or period of unidirectional grooming towards a recipient who is not engaged in grooming, that is, the engagement of one interactant given towards a recipient/other interactant within a grooming interaction (Foster et al. 2009). The grooming phase may also be mutual (overlapping grooming roles, e.g., A grooms B and B grooms A). The ending phase of an interaction was defined as the time point when either the interactant ceased grooming, engaged in alternative activities, including resting, for more than 30 s (Newton-Fisher and Lee 2011), or performed leave-taking actions.

### Coding

From these interactions, the identities of the interactants, dyad, and interaction were noted, and we coded the four following parameters. First, we coded gestures (characterised as the signals) and actions (see Supplementary Material and Table S1 for definitions and details of each gesture and action). Second, we coded turn transitions. Following Hilbrink et al. (2015), turn transitions were operationalised as a unit (signal/gesture or action, turn one) produced by one individual and receiving a unit (signal/gesture or action) from another individual, involving role reversal (signaler/actor-recipient roles). Turn transitions could range from none (i.e., unidirectional grooming interaction) to multiple within a single grooming interaction, so we also counted the frequency of turn transitions per interaction. To not only predict possible outcomes but also assess turn transition data for potential biases, we examined their frequency to ensure a comprehensive and robust approach. Third, we coded turn transition types, which could be action – action, action – signal, signal – action, or signal – signal. Fourth, we coded temporal relationships (timings) between the units in turn transitions, specifically from the offset (end timestamp) of a unit to the onset (beginning timestamp) of the following unit within 30 s. Overlap avoidance represents response latencies that have negative timing values, whereas positive timing values represent response latencies that involve overlap. We additionally calculated the temporal relationships for each turn transition type: action – action, action – signal, signal – action, and signal – signal to be considered as associated (Newton-Fisher and Lee 2011; Roberts et al. 2012).

#### Inter-reliability

15% of all coded video clips were recorded by two additional coders who were blind to the research objectives, and inter-observer agreement was evaluated for each coded Tier using the EasyDIAg software package (Holle and Rein 2015). This programme facilitates the assessment of rater agreement in ELAN, which is determined through a composite measure of both the annotation value and duration of a coded segment between raters. To establish agreement, the duration of the coded segments was required to overlap by at least 60%, and the annotation values needed to be identical (Holle and Rein 2015). The results of the tests indicated a ‘good’ level of agreement between the raters (rater 1, Cohen’s κ = 0.81; rater 2, Cohen’s κ = 0.88) and the respective coder. In instances where agreement was not achieved, these cases were re-evaluated by the respective coder and discussed between the coder and the rater. Once agreement was attained, these instances were resolved.

### Data analysis

#### Dominance rank

To determine the dominance hierarchy, we used only dyadic interactions with a clear ‘winner’ and ‘loser’ from an aggressive interaction (Total N_central_=180, N_western_=69), along with two given submissive behaviours (Total N_central_=254, N_western_=81), pant-grunts (Bygott 1979; Noe et al. 1980), and pant bark vocalisations (Rosati et al. 2020). We then created an interaction matrix from this dataset and applied MatMan analysis (de Vries et al. 1993) in R to determine the ordinal ranking. Males were rated ordinally, with the highest-ranked male (the alpha male) receiving a value of 1 and lower-ranked males receiving numerically greater numbers. The MatMan analysis computes the improved index of linearity (h0) and accounts for tied and unknown relationships (de Vries 1995), whereby an index score of 0.90 or higher is an indication of a clear linear hierarchy (de Vries 1998). Linear hierarchies differ among different chimpanzee communities, where the level of despotism is not uniform across communities (Kaburu and Newton-Fisher 2015). In some communities, the gamma positions, in comparison to the alpha and beta positions, are not as clear (Nakamura et al. 2015), which may be because adolescent males do not form a clear dominance hierarchy (Bygott 1979; Sandel et al. 2017). Therefore, we applied a steepness measure to assess the distribution of dominance, whether it was equally shared (egalitarian) or concentrated among a few individuals (despotic). The steepness measure considers the differences in aggressive and submissive interactions across individuals and is based on normalised David’s score. It was implemented using the *steepness* package and steeptest function in R, resulting in a score between zero and one, with zero indicating an egalitarian dominance hierarchy and one representing a despotic dominance hierarchy.

#### Social bonds

To determine the strength of social bonds across dyads, we applied the Dyadic Composite Sociality Index (DSI) to determine the strength of social relationships (Silk et al. 2013). The DSI score was quantified through grooming frequencies (mean per dyad: N_central_= 3.03 ± 3.26, N_western_= 5.19 ± 5.80), extracted from the focal data, and spatial proximity frequencies extracted from the scan data (mean per dyad: N_central_= 4.46 ± 5.01, N_western_=7.62 ± 7.72) of the dataset. Spatial proximity patterns reflect the strength of relationships, whereas grooming patterns reflect the quality of the relationships (Mitani 2009). The DSI was calculated using the following equation:

DSI = $$\:\left(\frac{{G}_{A+B}}{{G}_{A+B}\:+\:{G}_{A}+\:{G}_{B}}\right)+\left(\frac{{P}_{A+B}}{{P}_{A+B}\:+\:{P}_{A}+\:{P}_{B}}\right)\:\:$$

G_A+B_ is the combined grooming frequency of individual A towards individual B and vice versa, B to A. G_A_ represents the total grooming frequency observed by individual A in the absence of individual B, and G_B_ represents the total grooming frequency by individual B in the absence of individual A. Similarly, P_A+B_ is the number of scans where individuals A and B were observed to be in proximity to each other (within one meter). P_A_ and P_B_ represent the total number of scans for individuals A and B, respectively, excluding the other individual (A or B). Higher values represent strongly bonded dyads that had more frequent interactions than the average dyad in their community. Low values represent weakly bonded dyads with infrequent interactions compared with the average dyad of the community.

#### Implemented models

In contrast to frequentist statistics, which calculate the probability of observing data under the null hypothesis, Bayesian statistics offer insights into the credibility of the parameters used given the observed data (Kruschke 2014; McElreath 2018). Therefore, a series of Bayesian generalised mixed models was implemented in R (version 4.4.2; R Core Team 2022) using the function “brm” from the *brms* package (Bürkner 2017) to test the predictions. Each model included four Markov chain Monte Carlo (MCMC) chains with 4000 iterations per chain and specified 1000 warm-up iterations, leading to 16000 posterior samples. For all models, weakly informative priors were used to prevent overfitting (van de Schoot et al. 2021) and reduce inferential errors. For inference, we calculated 89% credit intervals (upper and lower) from the posterior distributions and checked whether zero was included in this interval (Kruschke 2014; McElreath 2018). Herein, we considered that, if posterior distributions shifted substantially away from zero in one direction, as opposed to centering on zero (i.e., which would indicate the null expectation of posterior distributions), there was evidence for an effect in a specific direction (positive or negative).

For each model (see Supplementary Material for the general model formula, Figure S1), the fixed effects were initiator’s age, recipient’s age, initiator’s dominance rank, recipient’s dominance rank, social bond strength (DSI), and relatedness (0 “not related”/1 “related”). All fixed effects, except relatedness, were z-transformed to improve the interpretation (Schielzeth 2010). Based on our predictions, we included interactions between age and dominance rank (six pairwise interactions). Interactions that showed no effect were omitted from the final model. To account for possible community differences, we added community identity (central or western) as a control effect. To avoid pseudoreplication, the random effects were the ID of the initiator, recipient, dyad and interaction.

To interpret the strength and uncertainty of the estimated effects, the median estimate and the median absolute deviation (MAD) were reported, along with the 89% Bayesian credible interval (89% CrI) and the probability of direction (pd). The probability of direction (positive or negative) ranges from 50% (high uncertainty about the direction) to 100% (strong certainty about the direction), and indicates the certainty with which an effect goes in a particular direction (Makowski et al. 2019).

Model diagnostics was performed by examining the model summaries, which indicated that the posterior distributions accurately represented the original response values. The R-hat statistics were deemed acceptable, with values of < 1.05. Furthermore, we confirmed adequate effective sample sizes (Bulk_ESS and Tail_ESS) of > 1000, and no divergent transitions in the MCMC chains. We also assessed model convergence by visually inspecting trace plots, histograms of posterior distributions, and checking for autocorrelation between iterations (see Supplementary Figure S2-S10; Depaoli and van de Schoot 2017).

##### Model 1: influence of demographic and social factors on turn transitions

To test whether demographic and social factors affected the likelihood of turn transitions, every occurrence of unit (signal/action) production (*N* = 6334) across all interactions was included as a data point. The response variable included whether there was an occurrence (binomial: yes/no) of a turn transition (fitting a Bernoulli distribution), and whether the produced unit entailed a consequent unit produced by the recipient. The interaction ID was added as a random effect.

##### Model 2: influence of demographic and social factors on the frequency of turn transitions

To test whether demographic and social factors influenced the frequency of turn transitions in a given interaction, each interaction (*N* = 311) was considered a data point, where the frequencies of turn transitions were tallied per interaction. The response variable was the number of turn transitions (fitting a Poisson distribution). The log of the total dyadic interaction time was integrated as an offset term (to account for the number of turn transitions relative to the interaction time). The interaction ID was added as a random effect.

##### Model 3: influence of demographic and social factors on turn transition types

To test whether demographic and social factors affected the likelihood of different turn transition types, a multivariate response model was developed using each turn transition (*N* = 2275) as an individual data point. In this model, each turn transition type was transformed into a binomial variable by converting each type into a numerical variable within the data frame and subsequently aggregating them to form the response variable. This approach allowed each turn transition type to be treated as a distinct binary variable in the same model. The interaction ID was added as a random effect.

##### Model 4: influence of demographic and social factors on temporal relationships

To test whether demographic and social factors influenced temporal relationships, each turn transition was considered a data point (*N* = 2275). The timings were log-transformed to normalise the data and reduce heteroscedasticity, and were input as the response variable (fitting a Gaussian distribution). Sum-to-zero contrast was applied prior to the turn transition types to facilitate interpretation and obtain the grand mean as the reference level instead of one of the four levels (action – action, action – signal, signal – action, and signal – signal). The turn transition types were additionally added as a fixed effect in interaction with all other fixed effects, to account for the influence of demographic and social factors on the temporal relationships across each type of turn transition.

## Results

Our analysis of the 311 grooming interactions across 157 dyads yielded 2275 turn transitions out of 6334 occurrences of signals or actions. Among these interactions, 275 involved at least one turn transition (*N* = 19 interactions; 0.07% with only one turn transition). Of the 2275 turn transitions, 38% were classified as action – action (*N* = 872), 32% as signal – action (*N* = 732), 16% as action – signal (*N* = 353), and 14% as signal – signal (*N* = 318). Furthermore, in 47% of the 2275 turn transitions there was overlap avoidance. In particular, action – action turn transitions (median = -0.14 s) were predominantly characterised by overlap avoidance, whereas overlap was mainly observed in action – signal (median = 0.10 s), signal – action (median = 0.06 s), and signal – signal (median = 0.74 s) turn transitions.

### Dominance rank and social bonds

The dominance hierarchy of the two communities revealed a significant dominance structure. However, ranks within this hierarchy were not strictly defined (linearity index: central: *h*’ = 0.417, *p* = 0.001; western: *h*’ = 0.355, *p* = 0.047), with the *h*’ index being less than 0.90 for both communities. The slope steepness (central: 0.17, *p* < 0.001; western: 0.19, *p* < 0.001), in line with the dominance hierarchy, suggested that the rank differences between consecutive positions in both communities were relatively small (e.g., no strong dominance between positions 4 and 5). These results indicate modest power differences between adjacently ranked individuals, and thus, a more egalitarian style in this population at the time of the study. The DSI values across 157 dyads from both communities ranged from 0 to 0.2069.

### Demographic and social factors on the likelihood and frequency of turn transitions

**Fig. 1:**
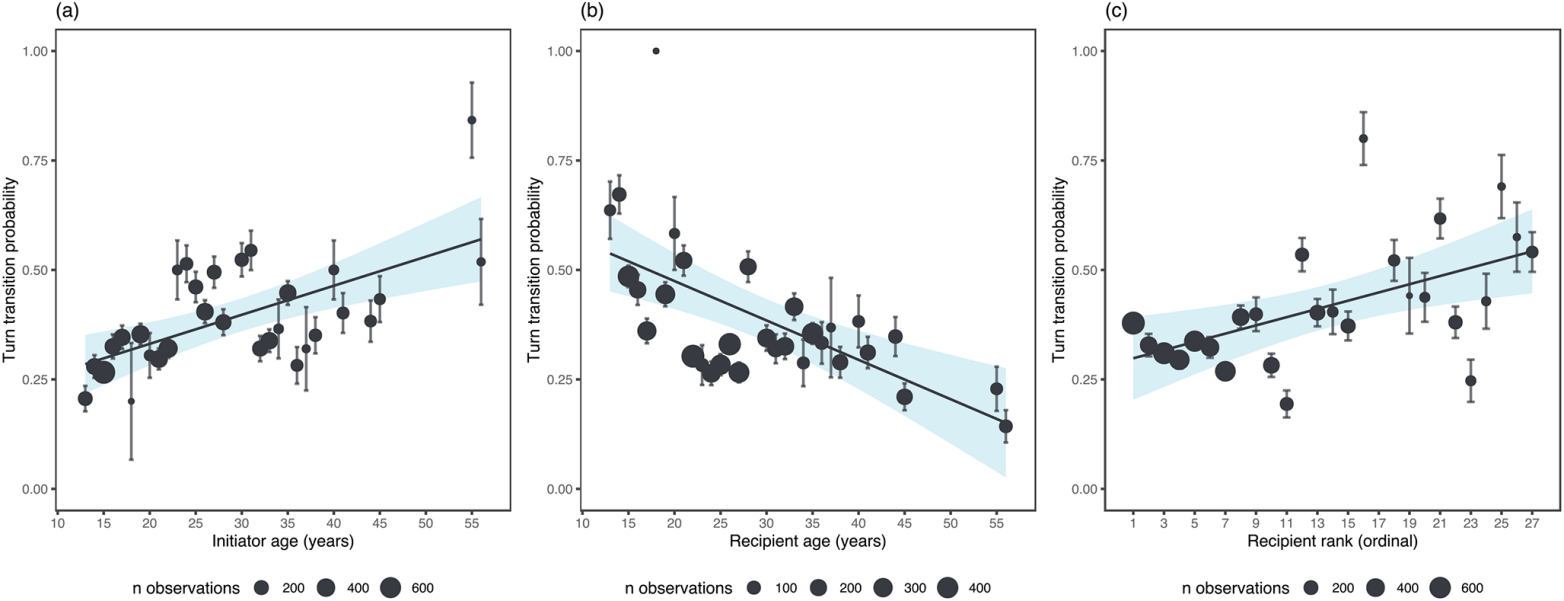
The plots display the strong effects from Model 1, showing the predicted probability of turn transitions in relation to (**a**) initiator’s age, (**b**) recipient’s age, and (**c**) recipient’s rank. The error bars represent the standard error, and the circles indicate the means, with their size reflecting the number of observations. The lines represent a linear regression model fit, and the shaded area indicates the confidence interval

Model 1 showed that initiator’s age, recipient’s age, and recipient’s rank influenced the likelihood of turn transitions (Fig. 1; see Supplementary Material Table S3). Turn transitions were more likely when they were initiated by an older individual (estimate [MAD] = 0.30 [0.08], 89% CI [0.20, 0.41], pd = 100%), when they involved younger recipients (estimate [MAD] = − 0.34 [0.09], 89% CI -0.46, -0.23], pd = 100%), and when recipients had a lower rank (estimate [MAD] = 0.20 [0.09], 89% CI 0.09, 0.32], pd = 98.01%). The interaction between initiator’s age and recipient’s rank showed strong evidence of an effect (estimate [MAD] = 0.10 [0.04], 89% CI 0.04, 0.15], pd = 98.61%). However, the social bond strength and relatedness did not influence the likelihood of turn transitions. These findings are partially support the prediction, where our findings confirmed that turn transitions are more likely when initiated by older individuals and when recipients are lower-ranking and younger. However, our findings did not support the prediction that turn transitions are more probable when initiated by higher-ranking individuals, and between strongly bonded dyads.

**Fig. 2:**
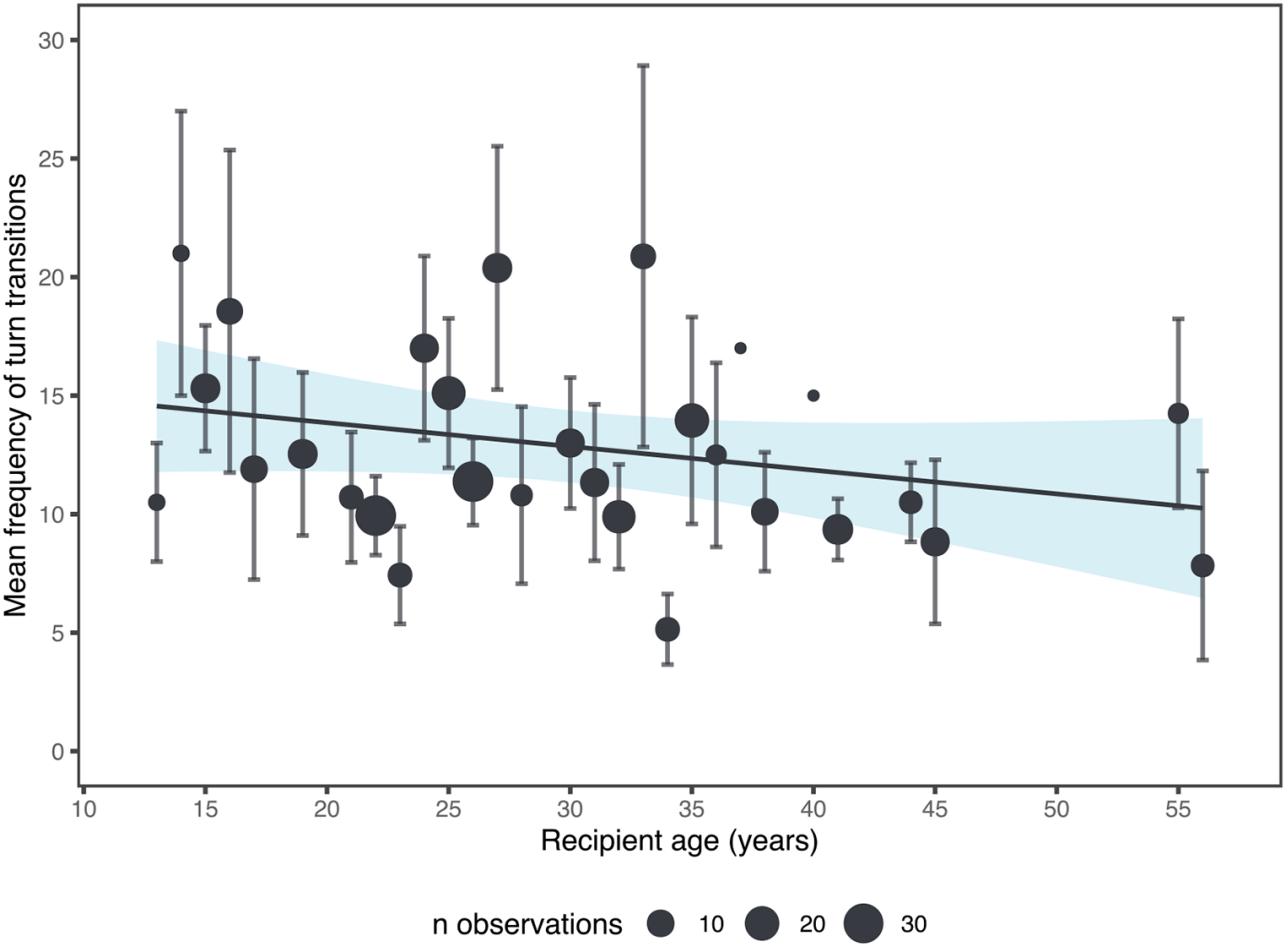
The plot displays the strong effects from Model 2, showing the frequency of turn transitions in relation to recipient’s age. The error bars represent the standard error, and the circles indicate the means, with their size reflecting the number of observations. The lines represent a linear regression model fit, and the shaded area indicates the confidence interval

Model 2 showed that recipient’s age had an impact on the number of turn transitions (Fig. 2, see Supplementary Material Table S4). Specifically, turn transitions were more frequent when recipients were younger (estimate [MAD] = − 0.11 [0.05], 89% CI -0.16, -0.05, pd = 66.61%). However, the pd was closer to 50%, suggesting relatively weak evidence for the direction of the effect. The other effects had no influence on the frequency of turn transitions (see Supplementary Material, Table S3). In relation to our prediction, despite the weak evidence of the effect and finding no influence of the initiator, we find partial support for the prediction that turn transitions are more frequent when involving younger recipients, but no support that recipients are of lower-rank.

### Demographic and social factors impact on turn transition types

**Fig. 3:**
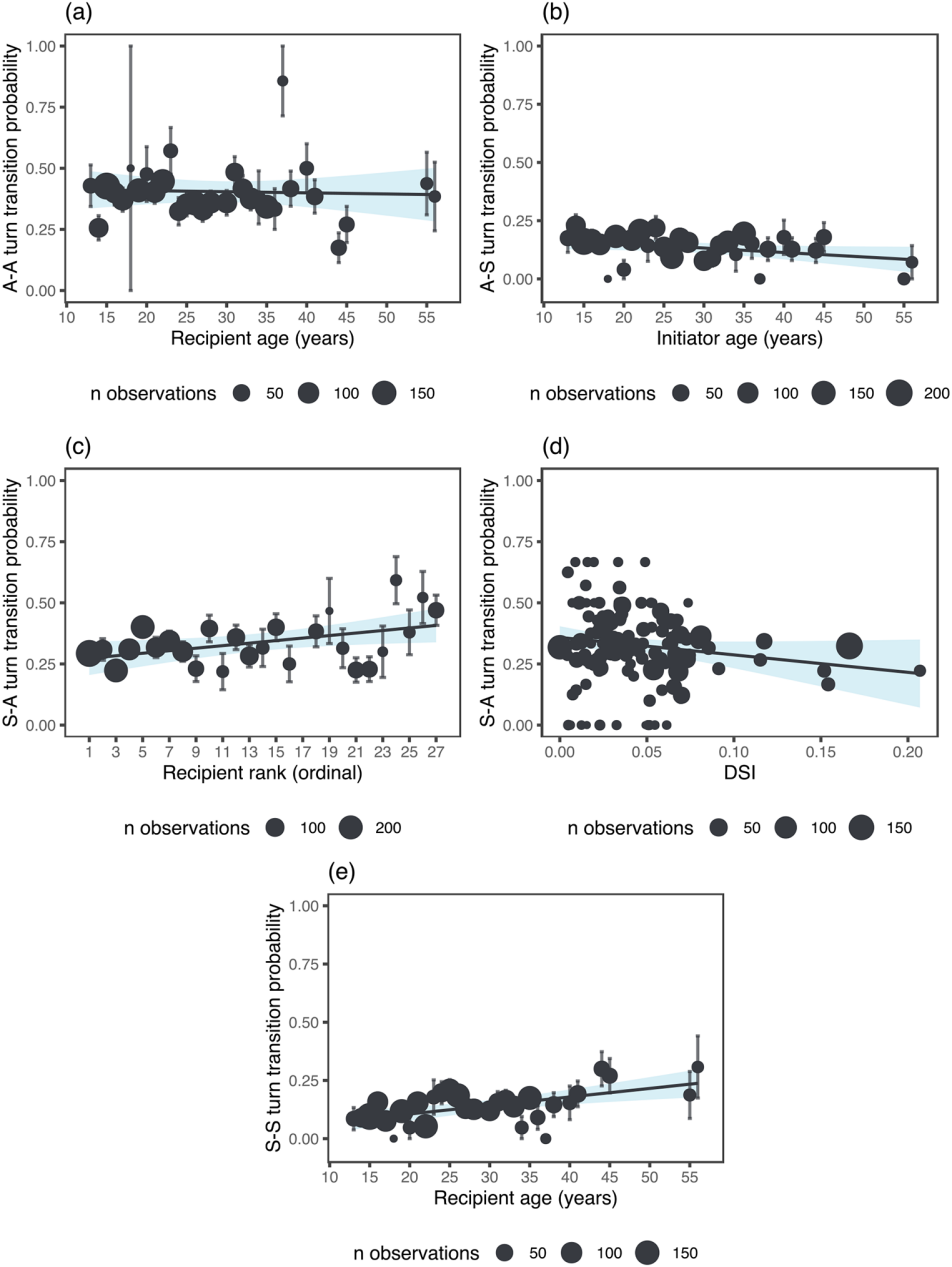
Plots display the strong effects from Model 3. Each plot showing the predicted probability of (**a**) action – action turn transitions in relation to recipient’s age, (**b**) action – signal turn transitions in relation to initiator’s age, signal – signal turn transitions in relation to (**c**) recipient’s rank and (**d**) social bond strength (DSI), and signal – signal turn transitions in relation to recipient’s age. The error bars represent the standard error, and the circles indicate the means, with their size reflecting the number of observations. The lines represent a linear regression model fit, and the shaded area indicates the confidence interval

Model 3 showed that there was an effect of age, dominance rank, and social bond strength across the turn transition types. Specifically, action – action turn transitions were more likely when the recipient was younger (estimate [MAD] = -0.12 [0.07], 89% CI [-0.20, -0.03], pd = 94.60%; Fig. 3a). Action – signal turn transitions were more probable when the initiator was younger (estimate [MAD] = -0.11 [0.09], 89% CI [-0.22, -0.01], pd = 90.65%; Fig. 3b). Signal – action turn transitions were more probable when they involved lower-ranking recipients (estimate [MAD] = 0.13 [0.09], 89% CI [0.02, 0.24], pd = 92.61%; Fig. 3c) and dyads with weaker bonds (estimate [MAD] = -0.11 [0.08], 89% CI [-0.20, -0.01], pd = 91.61%; Fig. 3d). Signal – signal turn transitions were more probable when they involved older recipients (estimate [MAD] = 0.26 [0.09], 89% CI [0.15, 0.37], pd = 99.86%; Fig. 3e). The other fixed effects did not influence the likelihood of turn transition types (see Supplementary Material, Table S5). In addition, action – action turn transitions were less probable in the western community (estimate [MAD] = -0.32 [0.20], 89% CI [-0.57, -0.07], pd = 94.29%), whereas signal – action turn transitions were more probable in the western community (estimate [MAD] = 0.30 [0.21], 89% CI [0.05, 0.56], pd = 92.21%). Our findings partially support our predictions. The finding that lower-ranking recipients were more likely to be involved in signal – action turn transitions, and younger recipients in action–action turn transitions supports the prediction that younger individuals and those with lower rank are more likely to give responses. However, our results that signal – signal turn transitions were more likely when they involved that older recipients and action – signal transitions were more likely when they involved younger initiator’s, showed the opposite to our prediction that turn transitions are more probable with adult initiators and younger recipients. Additionally, we predicted that dyads that are related and those that have stronger social bonds would have a higher likelihood of turn transitions, but this was not supported, as weakly bonded dyads were more likely to engage in signal – action transitions and there was no effect of relatedness.

### Demographic and social factors impact on temporal relationships

Model 4 showed no effect of demographic and social factors on the offset – onset timings (see Supplementary Material Table S6). Our prediction that social and demographic factors might influence the temporal relationship, specifically that overlap avoidance would be more likely when turn transitions involved adult recipients, was not supported by our findings.

## Discussion

This study aimed to assess the impact of different demographic and social factors on nonvocal turn-taking in non-human primates. We focused on male chimpanzee interactions in the cooperative context of grooming, to investigate the impact of age, dominance rank, relatedness, and social bonds on the (i) likelihood of turn transitions, (ii) frequency of turn transitions, (iii) likelihood of turn transition types, and (iv) temporal relationships. We predicted an increase in the likelihood of turn transitions when these were initiated by higher-ranking individuals, and adults as these individuals can have or have higher social standing and might be more inclined to receive responses, especially by recipients of lower rank and younger age. Furthermore, that the turn transition likelihood would be more probable between strongly bonded dyads and related dyads. Hence, we predicted adult recipients would more likely avoid temporal overlaps. Overall, our findings showed that turn transitions and transition types in grooming interactions were influenced by demographic and social factors, but not by relatedness. None of these factors had an effect on temporal relationships. These findings partially support the predictions of this study. In the following sections, we discuss these results in detail.

### The role of demographic and social factors on turn transitions

Our results showed that initiator’s age affected the likelihood of turn transitions contingent on the dominance rank of the recipient and vice versa, and the age of the recipient affected the likelihood of turn transitions. This finding may be due to the age and dominance rank of chimpanzee males being linked to each other (Rodrigues et al. 2022), where adolescent males are unequivocally subordinate to adult males (e.g., Hayaki 1990; Sandel et al. 2017), and senior males are subordinate to adult males (e.g., Baker 2000; Hayaki et al. 1989). Our findings further support this observation, with turn transitions being more probable when older individuals initiated a turn involving lower-ranking males, and when turn transitions involved younger recipients (i.e., adolescents). Thus, younger and lower-ranking individuals (if the initiator was older) were more likely to respond. It is possible that lower-ranking males exchange behaviours such as grooming to acquire social knowledge (e.g., identifying potential allies for social integration), in line with the *Biological Market Theory* (Noë and Hammerstein 1994, 1995). This theory posits that interactions between individuals can be conceptualised as a market, where interactions possess intrinsic value and individuals are regarded as traders whose selection of social partners is contingent upon the benefits these partners may provide. In this line, a study on eastern chimpanzees showed that older males engaged in aggressive interactions with conspecifics less frequently than other age classes (Rosati et al. 2020), suggesting that responding to these individuals is less likely to result in a possible aggressive interaction for lower-ranking males. Additionally, as the dominance of the two communities of the Ngogo population at the time of the study was not steep, agonistic support may not have been an important commodity (Kaburu and Newton-Fisher 2015). Thus, initiator dominance rank may not be relevant, explaining the possible lack of an effect of this factor. The higher likelihood and frequency of turn transitions with younger recipients can be attributed to adolescents’ tendency to interact more with older individuals, as observed in a previous study on the Ngogo population (Sandel et al. 2020). These young individuals may be attempting to build connections with older males rather than with their peers, as a means of gradually integrating themselves into the adult male dominance hierarchy. This is consistent with previous studies on grooming interactions in eastern chimpanzees, providing evidence that adolescents (younger individuals) interact selectively with adult males rather than with individuals of a similar age or rank (Hayaki 1988; Kawanaka 1993; Pusey 1990). In relation to turn-taking, our findings mirror the results of studies on call exchanges in other primate species. For example, calls from older individuals are more likely to elicit vocal responses both in captive Campbell monkeys (Lemasson et al. 2010) and captive common marmosets (*Callithrix jacchus*; Chen et al. 2009). Concerning dominance rank, studies have shown that dominant individuals received relatively more responses than subordinate ones as for example in captive and wild white-faced capuchins (Digweed et al. 2007) and captive western lowland gorillas (*Gorilla gorilla gorilla*; Lemasson et al. 2018).

The lack of an effect of social bonds and relatedness on the likelihood of turn transitions suggests that chimpanzees may prioritise immediate social needs (e.g., access to resources) over long-term affiliative relationships or genetic relatedness. This indicates that turn transitions may be driven more by short-term interactional goals rather than by maintaining long-term social bonds, which can be associated with relatedness, as shown in maternal brothers and unrelated males in eastern chimpanzees (e.g., Bray and Gilby 2020; Langergraber et al. 2007; Mitani 2009). Hence, social bonds and relatedness may have an impact on groom – groom turn transitions (i.e., a form of action – action turn transition), as shown previously in the Ngogo population (e.g., Mitani et al. 2000). However, grooming decisions may not be solely predicated on social bonds; the bonding model as an explanation for grooming in chimpanzees may not consistently apply (see Kaburu and Newton-Fisher 2016), which may additionally hold for turn transitions. Consequently, individuals may make choices based on their local markets (e.g., partner choice out of the present bystanders), where a markets-based model from the *Biological Market Theory* may be an important model for consideration (Kaburu and Newton-Fisher 2016). Nonetheless, this study examined various turn transitions beyond groom – groom transitions (a form of action – action turn transitions), where social bonds only had an effect on signal – action turn transitions. Thus, our findings on the impact of social and demographic factors on the likelihood of turn transitions may be further explained by the specific type of turn transition employed, with certain factors potentially exerting a more crucial influence on particular turn transition types, as highlighted in the following section.

Although research on the impact of demographic and social factors on conversational turn-taking in humans is limited and often refers to age in terms of development between mother-infant interactions (e.g., Bateson 1975; Gratier et al. 2015), our findings suggest that interacting partners can play a role in turn-taking, in line with the *Communication Accommodation Theory*, which may also apply to humans. Thus, although human conversational turn-taking and its underlying features have been proposed to be universal across languages and cultures (Kendrick et al. 2020; Levinson 2006; Stivers et al. 2009), there may still be an influence of whom one is turn-taking with.

### The role of demographic and social factors impact on turn transition types

From our results, we found that action – action turn transitions were more likely when they involved a younger recipient. This may be explained by the fact that younger individuals are more likely to direct one-sided grooming to conspecifics, i.e., they groom a conspecific regardless of reciprocation (Nishida 2011; Sandel et al. 2020; Watts 2000a). Similar to the likelihood of turn transitions, younger individuals may do so to establish and nurture social bonds or relationships with older, more dominant group members. This is also the time when adolescent males are observed to shift from interacting with their mothers to interacting with group members such as adult males (Pusey 1990). Additionally, individuals might prefer to interact with younger individuals, as they could be potential future allies, a pattern consistent with the framework of the *Biological Market Theory* (Noë and Hammerstein 1994, 1995).

For action – signal turn transitions, initiators were more likely to be younger; thus, younger individuals were more likely to use an action towards another. This result may be due to younger individuals being more likely to approach or groom others, in contrast to older individuals who may become more passive or selective in interactions, as observed in other eastern chimpanzee populations (Nakamura et al. 2015; Rosati et al. 2020).

Signal – action turn transitions may represent request – grant pairs (Pika et al. 2018), with findings indicating that younger individuals are more likely to respond to signals from other males. A possible explanation for this may be the tendency of younger males to position themselves for having interactions, by for instance joining subgroups with older males (Sandel et al. 2020) to establish social bonds and navigate complex social hierarchies. This suggests that younger males may exhibit a greater need to engage with others than their older counterparts. Furthermore, these signal – action turn transitions were more likely to occur between weakly bonded interactants. This may be explained by the fact that, prior to initiating an interaction with a weakly bonded partner, an initiator may signal a request to monitor whether the recipient is willing to engage in a grooming interaction. Engaging in such interactions may foster social tolerance, establish bonds between the involved individuals, and enhance the likelihood of receiving benefits, such as food sharing or support during conflicts. Hence, during a grooming interaction, weakly bonded interactants may need to request grooming to be reciprocated in comparison to strongly bonded dyads, where it may occur more naturally (e.g., Bray and Gilby 2020; Mitani 2009).

Concerning signal – signal turn transitions, these may reflect social negotiation, where individuals exchange signals with distinct meanings to initiate and achieve a desired outcome. In this study, negotiation refers to opposing intentions (e.g., both requesting to be groomed) where one individual achieves their desired outcome (i.e., receiving grooming), but the grooming interaction overall continues. We found that these turn transitions were more likely to occur when the recipient was older. This finding could be explained by older individuals being socially integrated (e.g., Nakamura et al. 2015) and holding a position that better allows them to negotiate (i.e., receiving rather than giving grooming), in comparison with younger individuals who are still growing into their social network (e.g., Nakamura et al. 2015). Thus, older chimpanzees may have more experience and social knowledge, which allows them to use grooming more effectively as a social tool. These older individuals are more experienced in choosing successful gestures, as posited by the *Repertoire Tuning hypothesis* (Hobaiter and Byrne 2011). This suggests that older chimpanzees have learned to negotiate grooming interactions more efficiently over time, and these signal – signal turn transitions are therefore more likely to involve older males.

Despite the interconnectivity of grooming and social bonds across chimpanzee populations (Mitani 2009; Muller and Mitani 2005; Newton-Fisher 2002; Watts 2002), social bonds only showed an effect on signal – action turn transitions, suggesting that may play a unique role in the translation of signals into actions during grooming interactions. Hence, mutual grooming (i.e., overlapping turns of grooming) is frequent in chimpanzees (e.g., Arnold and Whiten 2003; Boesch and Boesch-Achermann 2000; Gomes et al. 2009; Nakamura 2003; Reynolds 2005) and is likely linked with social bonds (Fedurek et al. 2009; but see Machanda et al. 2014). However, mutual grooming entail overlapping turns and we found that action – action turn transitions abided to the overlap avoidance, where groom – groom turn transitions (a form of action – action turn transitions) may rarely have been mutual grooming. Although our study looked at turn transitions in general and not at the specific units of turn transitions, if mutual grooming is connected with social bonds, this may explain why our findings show a lack of effect of social bonds, particularily regarding the action – action turn transitions. This finding may additionally be explained by the fact that dominance rank also plays a significant role in the social bonding and relationships of chimpanzees (e.g., Bray et al. 2021; Watts 2000b), and age has been shown to correlate with rank (e.g., Hayaki 1990; Hayaki et al. 1989). Thus, social bonds in chimpanzees can be considered to be multifaceted in relation to grooming. Therefore, it may still provide support for the “*grooming to gossip*” hypothesis (Dunbar 1996, 2004), where turn-taking in grooming interactions may represent an early behavioural framework for coordinating social interactions to effectively bond, which later evolved into the turn-taking infrastructure fundamental to human language.

Relatedness had no influence on any turn transition type, suggesting that genetic closeness may not be a determining factor in the dynamics of turn-taking in grooming. This can be explained by the fact that relatedness possibly plays a more crucial role during individual development, such as between mother and infant pairs in chimpanzees (e.g., Fröhlich et al. 2016), as seen in humans during “protoconversations” (Gratier et al. 2015).

### The role of demographic and social factors impact on temporal relationships

The demographic and social factors tested in this study had no effect on offset – onset timings. This may imply that regardless of whom a male chimpanzee in the Ngogo population is interacting with, temporal relationships are a universal feature of their grooming interactions. Whereby, the average timing found in this study matches that of other chimpanzee studies (e.g., Badihi et al. 2024; Fröhlich et al. 2016). This goes in line with human conversational turn-taking, where across ten languages in humans, temporal relationships are similar, suggesting a possible universal aspect (Stivers et al. 2009), although the interpersonal context role in these timings is still to be understood. In the future, more studies on other chimpanzee populations and subspecies will be necessary to confirm if the lack of demographic and social effects on offset – onset timings is truly universal.

## Conclusion

This study represents an initial investigation into the impact of demographic and social factors on nonvocal turn-taking in a non-human species, chimpanzees. Cooperative turn-taking has been proposed as an ancient underpinning of the layered language system (Levinson 2016); however, virtually no knowledge exists regarding the social function of turn-taking in human societies (Levinson 2016; Sacks et al. 1974) or the animal kingdom (Pika et al. 2018). Consequently, this study aimed to address this research gap. Our findings revealed an effect of age, dominance rank, and, to some extent, social bonds, but no effect of relatedness on the turn transition infrastructure. These results suggest that turn-taking during grooming reflects a distinct layer of social and communicative coordination that extends beyond grooming behaviours. Demographic and social factors shape how individuals accommodate their partners through reciprocal or asymmetrical exchanges, in alignment with the principles of the *Communication Accommodation Theory*. By conceptualising turn-taking as a behavioural accommodation mechanism, researchers can gain deeper insights into the intricate social dynamics and communication strategies employed by chimpanzees during grooming interactions. This approach will not only enhance our understanding of chimpanzee social cognition, but will also provide valuable perspectives on the evolutionary foundations of human communication (e.g., Dunbar 1996, 2004).

## Electronic supplementary material

Below is the link to the electronic supplementary material.

Supplementary Material 1

## Data Availability

Data used during this study is available at Figshare: 10.6084/m9.figshare.28302725.v1.

## Footnotes

1 Gestures are depicted in small capitals.
