# Supplementary material for: Turn-taking in grooming interactions of chimpanzees (Pan troglodytes schweinfurthii) in the wild: the role of demographic and social factors
Source: Anim Cogn. 2025 Mar 24;28(1):26. doi: 10.1007/s10071-025-01940-7 (PMC11933235; doi:10.1007/s10071-025-01940-7)
Supplement: Supplementary file 1 — Supplementary Material 1 [file 10071_2025_1940_MOESM1_ESM.docx]

**Turn-taking in grooming interactions of chimpanzees (*Pan troglodytes schweinfurthii*) in the wild: The role of demographic and social factors**

Kayla Kolff^1*^ & Simone Pika^1^

^1^Institute of Cognitive Science, Osnabrück University, Osnabrück, Germany

***Corresponding Author(s):**

Kayla Kolff: [kayla.w.t.kolff@gmail.com](mailto:kayla.w.t.kolff@gmail.com), ORCID: 0000-0001-8625-2185

**Supplementary Information**

**Table S1**. Gesture (depicted in small capitals) and action types produced during grooming interactions in chimpanzee male dyads identified in this study.

| Gesture/action | Definition (this study) | Modality |
| --- | --- | --- |
| exaggerated loud scratch (els) | Amplified scraping movement on signaller's own body, producing an amplified noise. If contact stops or there is a change in bodily location, it is recognized as a new els. | AV |
| extend limb | Signaller stretches their limb (usually an arm or a leg) horizontally towards or in view of the recipient. | V |
| grab-pull | The signaller's hand (1 or 2 handed) is firmly closed over part of the recipient's body or a handful of hair and a force exerted to move the recipient’s body part towards the signaller. | T |
| handclasp | One or both individuals raise one arm simultaneously above their heads and grasp a part of the arm of the other individual, either the palm, wrist, forearm, or elbow. | T |
| hold | The signaller's hand or foot is firmly closed over a part of the recipient's body or a handful of hair, similar to grab-pull but excludes the pulling and lasts for >2 seconds. | T |
| peer | The signaller maintains eye-contact with the recipient for a duration >2 seconds while being in close proximity to the recipient (within 1m). | V |
| present | The signaller moves their body or a body part to deliberately expose a specific area to the visual field of the recipient and the signaller maintains that position for > 2 seconds. | V |
| push | The signaller makes physical contact with the recipient's body or a body part (typically by using a hand or a foot) and applies some force to the body or the body part of the recipient. This gesture is not effective in achieving its goal (e.g., the desire that the recipient or a body part of his moves in a certain direction), but the voluntary movement is performed by the recipient to meet a specific goal. | T |
| raise | The signaller lifts one of their body parts (typically a hand, an arm, a head, or a leg) in a generally vertical movement, often by including a brief pause of a minimum of 1 second at the peak of the movement. raise hand entails only the lower part of the arm without the elbow being raised above the shoulder, while raise arm involves the whole arm being raised above the shoulder. | V |
| touch | Light and brief contact (<2 seconds) by the signaller (typically with the fingers, knuckles, hand, or foot) on the body or a body part of the recipient. | T |
| touch hold | Light contact is initiated or maintained by the signaller (typically fingers, knuckles, hand, or foot) on the body or body part of the recipient with contact maintained for >2 seconds. | T |
| Approach | An individual moves closer towards a recipient into a proximity within arm’s length (1m). |  |
| Display | A slow but exaggerated rhythmic movement including piloerection by an individual that builds towards directly or indirectly running at a fast speed towards another and can include drumming (hitting and/or kicking tree buttresses) or throwing/shaking objects. |  |
| Groom | Use of both hands, pushing the hair back with the thumb or index finger of one hand and holding it back while picking at the exposed skin with the nail of the corresponding thumb or index finger of the other hand. An individual can also use their mouth to hold back the hair with their lower lip while picking through the exposed skin. |  |
| Leave | An individual moves away from another one out of proximity. |  |
| Mount | An individual embraces another one with one or both arms from behind. |  |
| Move away | Minimal movement away from another individual and resting at a spot which is still within close proximity (arm’s length, approximately 1- 2m distance) to the other individual. |  |
| Reposition | An individual either stays in the same spot but adjusts their bodily position, for example, by shifting from a sitting position to a laying position without exaggerating a new bodily area (otherwise classified as a present), or a slight shift (<1m) from one spot to another while maintaining close proximity (close body contact). |  |

Based on existing ethograms and definitions of other studies (Goodall, 1986; Grund et al., 2023; Nishida et al., 1999; Pika & Mitani, 2006; Wilke et al., 2022). Gestures were categorized according to their modality where A refers to auditory, V to visual, and T to tactile components.

**Table S2.** Age of the focal subjects at the time of the study.

| **Community** | **Focal** | **Age** |
| --- | --- | --- |
| Central | ABR | 22 |
| Central | BOO | 19 |
| Central | BOS | 18 |
| Central | BRO | 55 |
| Central | CAN | 14 |
| Central | CAR | 30 |
| Central | CHO | 21 |
| Central | DJA | 25 |
| Central | DYL | 15 |
| Central | ELT | 17 |
| Central | EVA | 26 |
| Central | FLE | 15 |
| Central | GAR | 14 |
| Central | GUS | 33 |
| Central | HAD | 23 |
| Central | JAC | 32 |
| Central | LOV | 41 |
| Central | MIL | 23 |
| Central | MIT | 38 |
| Central | MOR | 33 |
| Central | MUL | 15 |
| Central | NEL | 22 |
| Central | PEE | 15 |
| Central | PET | 26 |
| Central | POW | 16 |
| Central | PUC | 13 |
| Central | WIS | 22 |
| Western | BAC | 13 |
| Western | BIL | 22 |
| Western | BUC | 17 |
| Western | DAM | 15 |
| Western | FRE | 13 |
| Western | GAI | 44 |
| Western | HUT | 28 |
| Western | LAR | 17 |
| Western | MUR | 14 |
| Western | RIM | 35 |
| Western | ROL | 35 |
| Western | WAY | 26 |
| Western | WES | 24 |
| Western | WIL | 14 |
| Western | YOY | 15 |

**Figure S1**. General model formula

*Response variable ~ initiator age (z-transformed)^[[1]](#footnote-1)^ + recipient rank (z-transformed) + recipient age (z-transformed) + initiator rank (z-transformed) + DSI (z-transformed) + relatedness + community ID +*

*(1|initiator) +*

*(1|recipient) +*

*(1|dyad_ID) +*

*(1|interaction_ID)^[[2]](#footnote-2)^*

**Table S3**. Turn transition probability (Model 1) testing the effect of demographic and social factors on the likelihood of a turn transition (N = 6334). Posterior estimates of the effect of demographic and social factors on the probability of turn transitions.

|  | **Median estimate** | **MAD** | **89% Crl**  **[lower, upper]** | **PD** |
| --- | --- | --- | --- | --- |
| Intercept | -0.69 | 0.15 | -0.87, -0.50 | 100% |
| ***Initiator age*** | ***0.30*** | ***0.08*** | ***0.20, 0.41*** | ***100%*** |
| ***Recipient age*** | ***-0.34*** | ***0.09*** | ***-0.46, -0.23*** | ***100%*** |
| Initiator rank | -0.10 | 0.10 | -0.22, 0.02 | 84.37% |
| ***Recipient rank*** | ***0.20*** | ***0.09*** | ***0.09, 0.32*** | ***98.01%*** |
| Social bonds | -0.02 | 0.06 | -0.09, 0.06 | 63.20% |
| Relatedness | 0.16 | 0.17 | -0.06, 0.38 | 81.22% |
| Community ID[WE] | 0.11 | 0.26 | -0.19, 0.44 | 67.17% |
| ***Initiator age:Recipient rank*** | ***0.10*** | ***0.04*** | ***0.04, 0.15*** | ***98.61%*** |

**
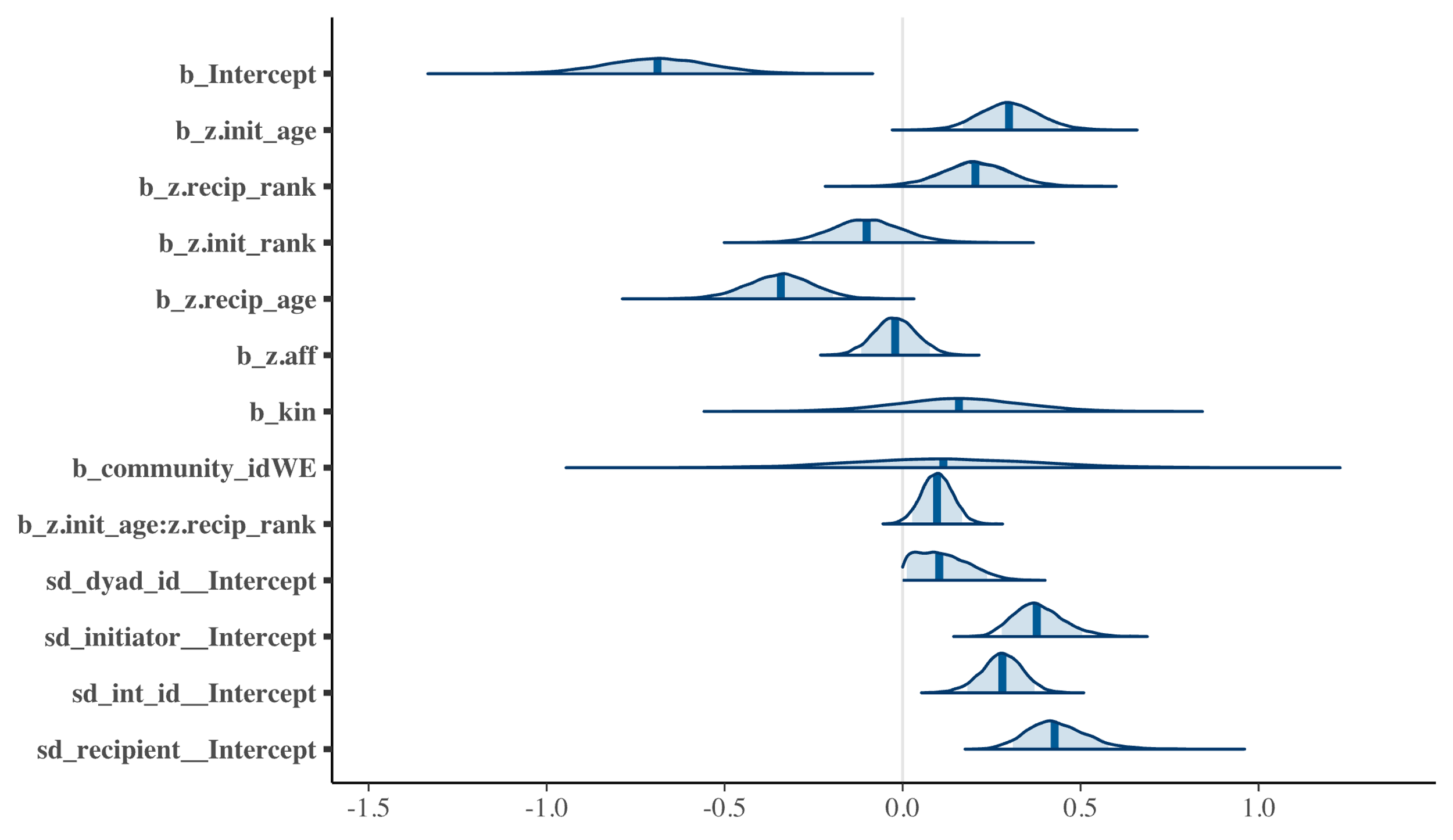
**

**Figure S2.** Density plot of posterior distributions and 89% Credible Intervals for Bayesian generalized linear mixed model 1.


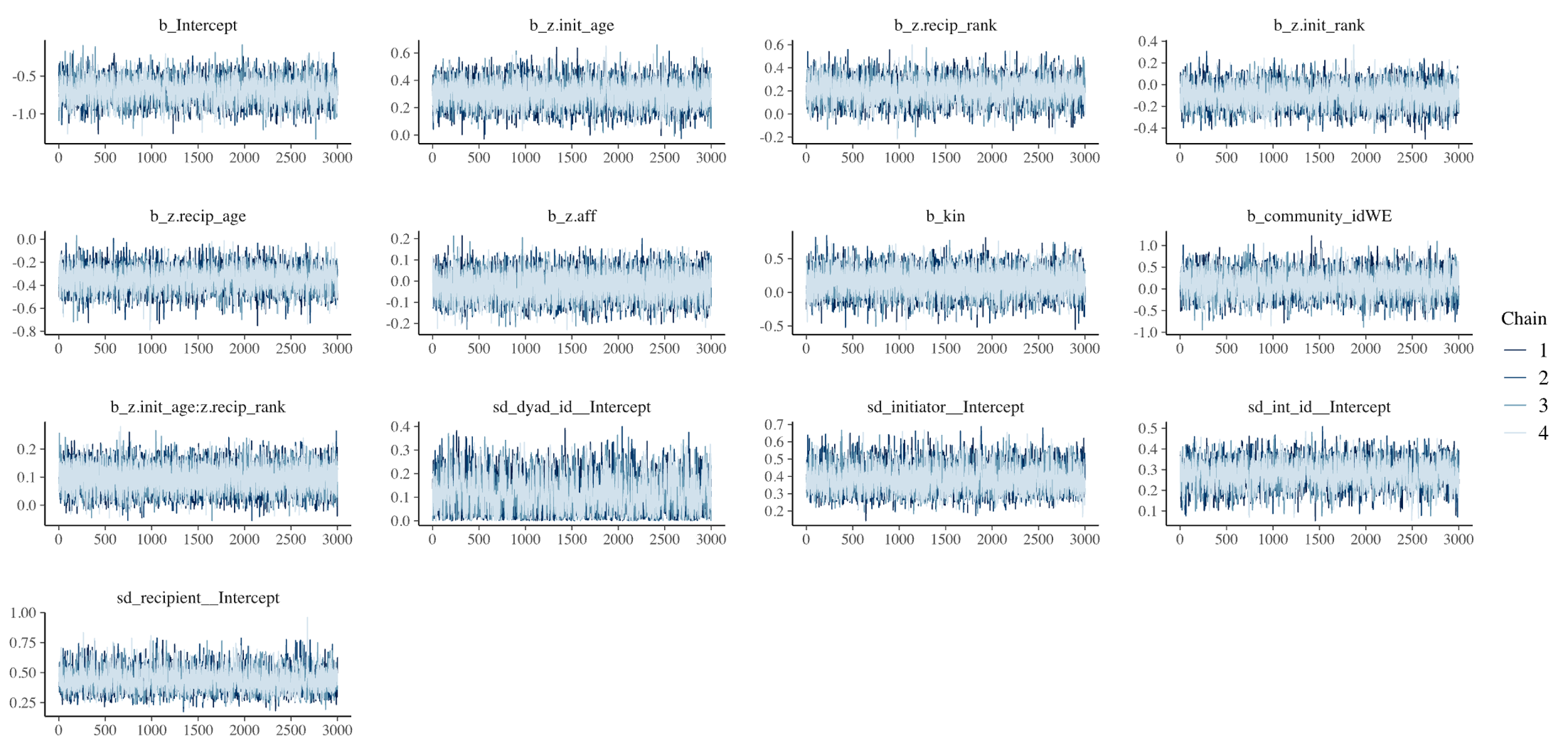


**Figure S3.** MCMC trace-plots of posterior distributions of the Bayesian generalized linear mixed model 1.

**Table S4**. Turn transition frequency (Model 2) testing the effect of dyadic demographic and social factors on the frequency of a turn transition for each interaction (N = 311). Posterior estimates of investigating the effect of demographic and social factors on the frequency of turn transitions.

|  | **Median estimate** | **MAD** | **89% Crl**  **[lower, upper]** | **PD** |
| --- | --- | --- | --- | --- |
| Intercept | -10.35 | 0.07 | -10.43, -10.26 | 100% |
| Initiator age | -0.01 | 0.06 | -0.10, 0.05 | 54.38% |
| ***Recipient age*** | ***-0.11*** | ***0.05*** | ***-0.16, -0.05*** | ***66.61%*** |
| Initiator rank | -0.02 | 0.06 | -0.10, 0.05 | 98.43% |
| Recipient rank | 0.02 | 0.05 | -0.04, 0.09 | 66.81% |
| Social bonds | 0.02 | 0.05 | -0.05, 0.08 | 61.20% |
| Relatedness | 0.10 | 0.17 | -010, 0.31 | 72.72% |
| Community ID | 0.08 | 0.12 | -0.07, 0.23 | 73.75% |

**
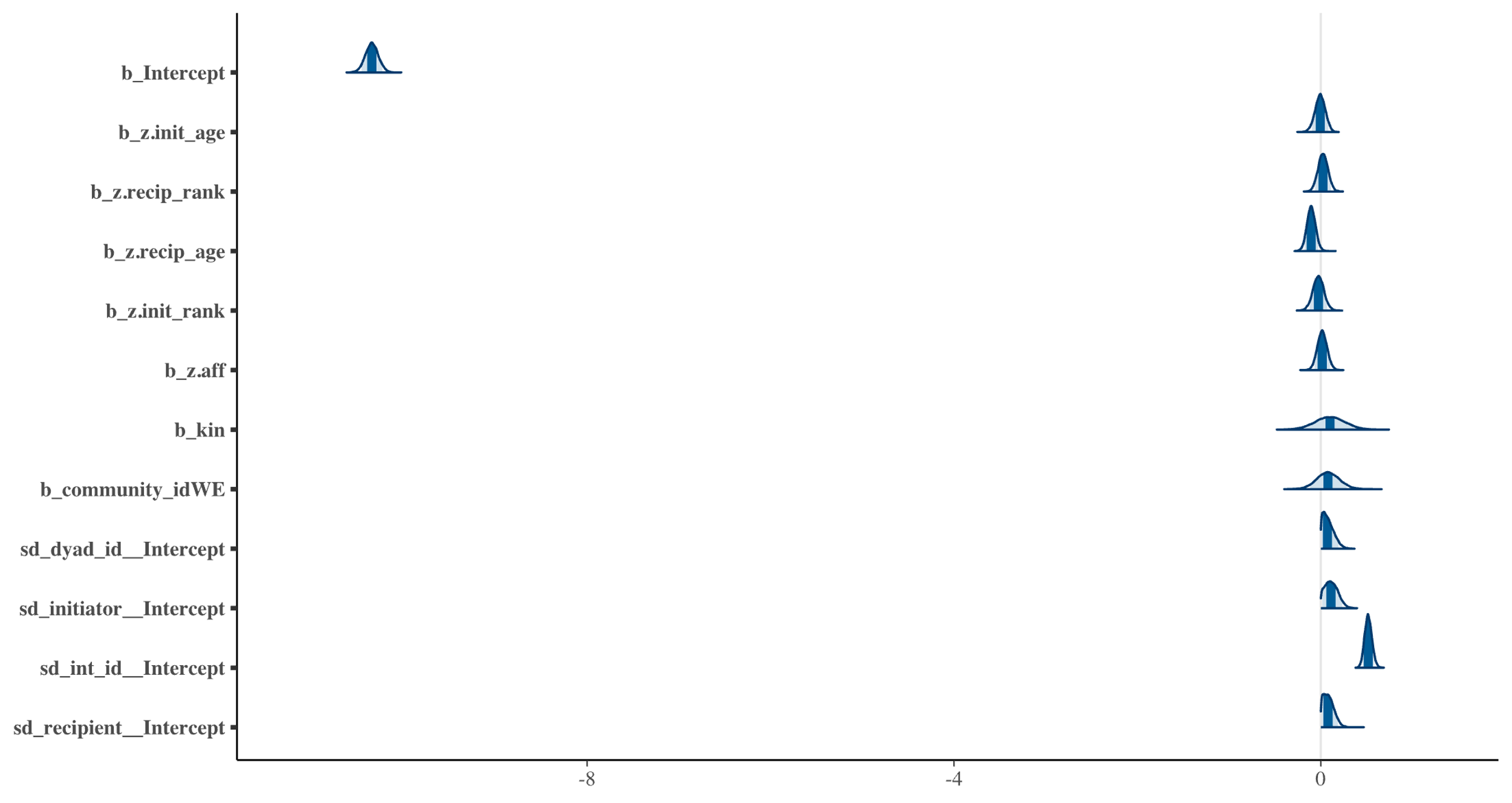
**

**Figure S5.** Density plot of posterior distributions and 89% Credible Intervals for Bayesian generalized linear mixed model 2.

**
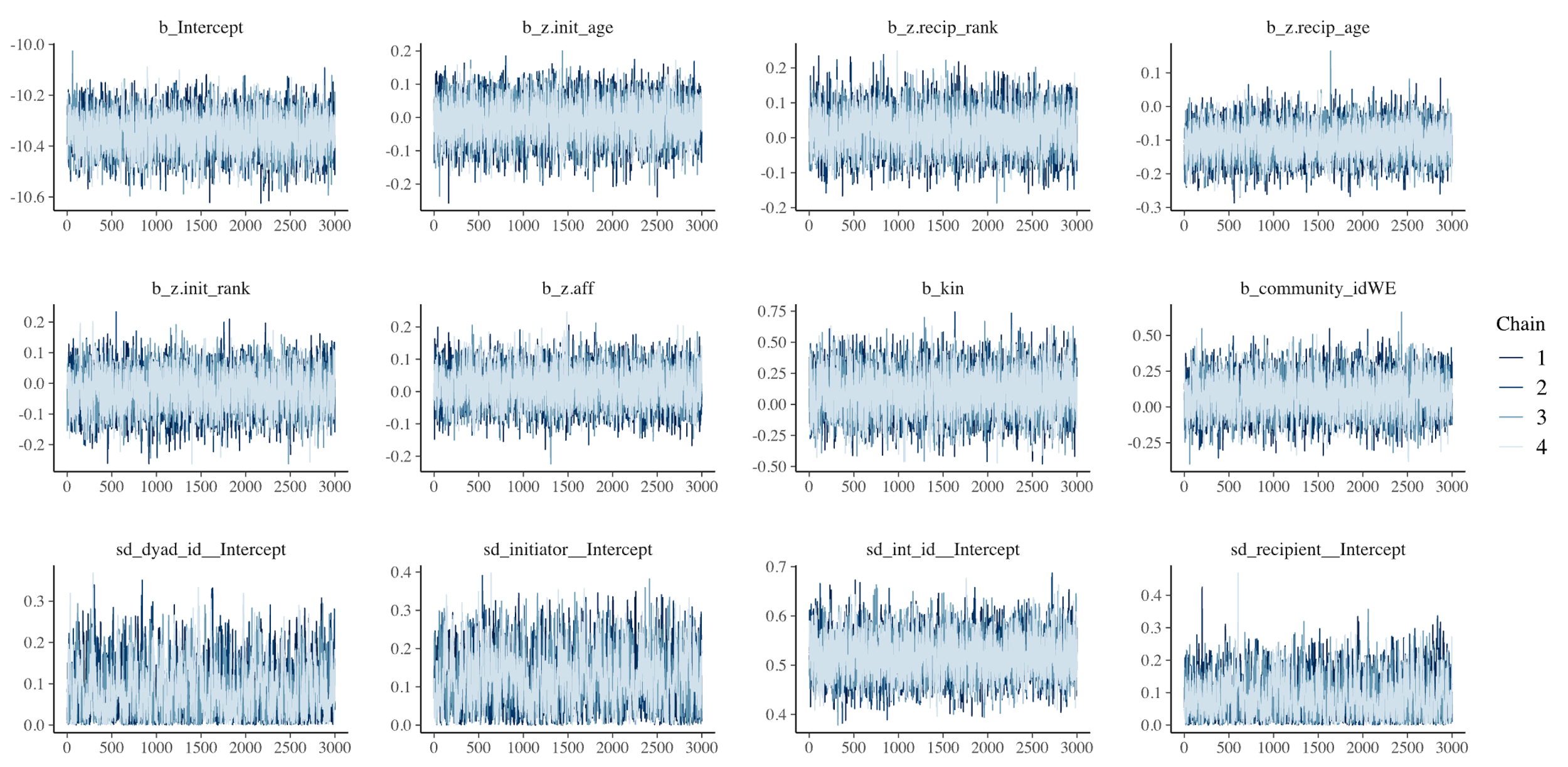
Figure S6.** MCMC trace-plots of posterior distributions of the Bayesian generalized linear mixed model 2.

**Table S5**. Turn transition type probability (Model 3) testing the effect of dyadic demographic and social factors on the turn transition type (N = 2275). Posterior estimates of the effect of demographic and social factors on the probability of the type of turn transitions.

|  | **Median estimate** | **MAD** | **89% Crl**  **[lower, upper]** | **PD** |
| --- | --- | --- | --- | --- |
| AA_Intercept | -0.33 | 0.11 | -0.47, -0.19 | 99.82% |
| AA_Initiator age | -0.02 | 0.08 | -0.11, 0.08 | 57.85% |
| ***AA_Recipient age*** | ***-0.12*** | ***0.07*** | ***-0.20, -0.03*** | ***94.60%*** |
| AA_Initiator rank | -0.05 | 0.09 | -0.16, 0.07 | 70.14% |
| AA_Recipient rank | -0.09 | 0.07 | -0.18, 0.00 | 88.95% |
| AA_Social bonds | 0.07 | 0.09 | -0.04, 0.19 | 78.13% |
| AA_Relatedness | 0.02 | 0.27 | -0.30, 0.36 | 52.66% |
| ***AA_Community ID [WE]*** | ***-0.32*** | ***0.20*** | ***-0.57, -0.07*** | ***94.29%*** |
| AS_Intercept | -1.68 | 0.12 | -1.82, -1.54 | 100% |
| ***AS_Initiator age*** | ***-0.11*** | ***0.09*** | ***-0.22, -0.01*** | ***90.65%*** |
| AS_Recipient age | -0.01 | 0.09 | -0.11, 0.10 | 54.78% |
| AS_Initiator rank | 0.08 | 0.10 | -0.04, 0.20 | 79.03% |
| AS_Recipient rank | -0.09 | 0.08 | -0.19, 0.01 | 86.89% |
| AS_Social bonds | 0.08 | 0.09 | -0.03, 0.20 | 81.91% |
| AS_Relatedness | -0.13 | 0.25 | -0.43, 0.18 | 69.59% |
| AS_Community ID [WE] | -0.03 | 0.21 | -0.28, 0.23 | 55.83% |
| SA_Intercept | -0.93 | 0.12 | -1.08, -0.79 | 100% |
| SA_Initiator age | 0.08 | 0.07 | 0.00, 0.17 | 88.14% |
| SA_Recipient age | -0.05 | 0.08 | -0.16, 0.05 | 72.27% |
| SA_Initiator rank | 0.05 | 0.08 | -0.05, 0.15 | 72.89% |
| ***SA_Recipient rank*** | ***0.13*** | ***0.09*** | ***0.02, 0.24*** | ***92.61%*** |
| ***SA_Social bonds*** | ***-0.11*** | ***0.08*** | ***-0.20, -0.01*** | ***91.61%*** |
| SA_Relatedness | 0.07 | 0.21 | -0.19, 0.33 | 62.19% |
| ***SA_Community ID [WE]*** | ***0.30*** | ***0.21*** | ***0.05, 0.56*** | ***92.21%*** |
| SS_Intercept | -2.07 | 0.14 | -2.24, -1.90 | 100% |
| SS_Initiator age | 0.03 | 0.09 | -0.08, 0.10 | 61.78% |
| ***SS_Recipient age*** | ***0.26*** | ***0.09*** | ***0.15, 0.37*** | ***99.86%*** |
| SS_Initiator rank | -0.05 | 0.11 | -0.18, 0.09 | 66.78% |
| SS_Recipient rank | 0.00 | 0.10 | -0.11, 0.12 | 50.24% |
| SS_Social bonds | -0.07 | 0.12 | -0.21, 0.09 | 70.39% |
| SS_Relatedness | 0.03 | 0.33 | -0.38, 0.44 | 53.73% |
| SS_Community ID [WE] | 0.28 | 0.23 | 0.00, 0.58 | 88.40% |

**
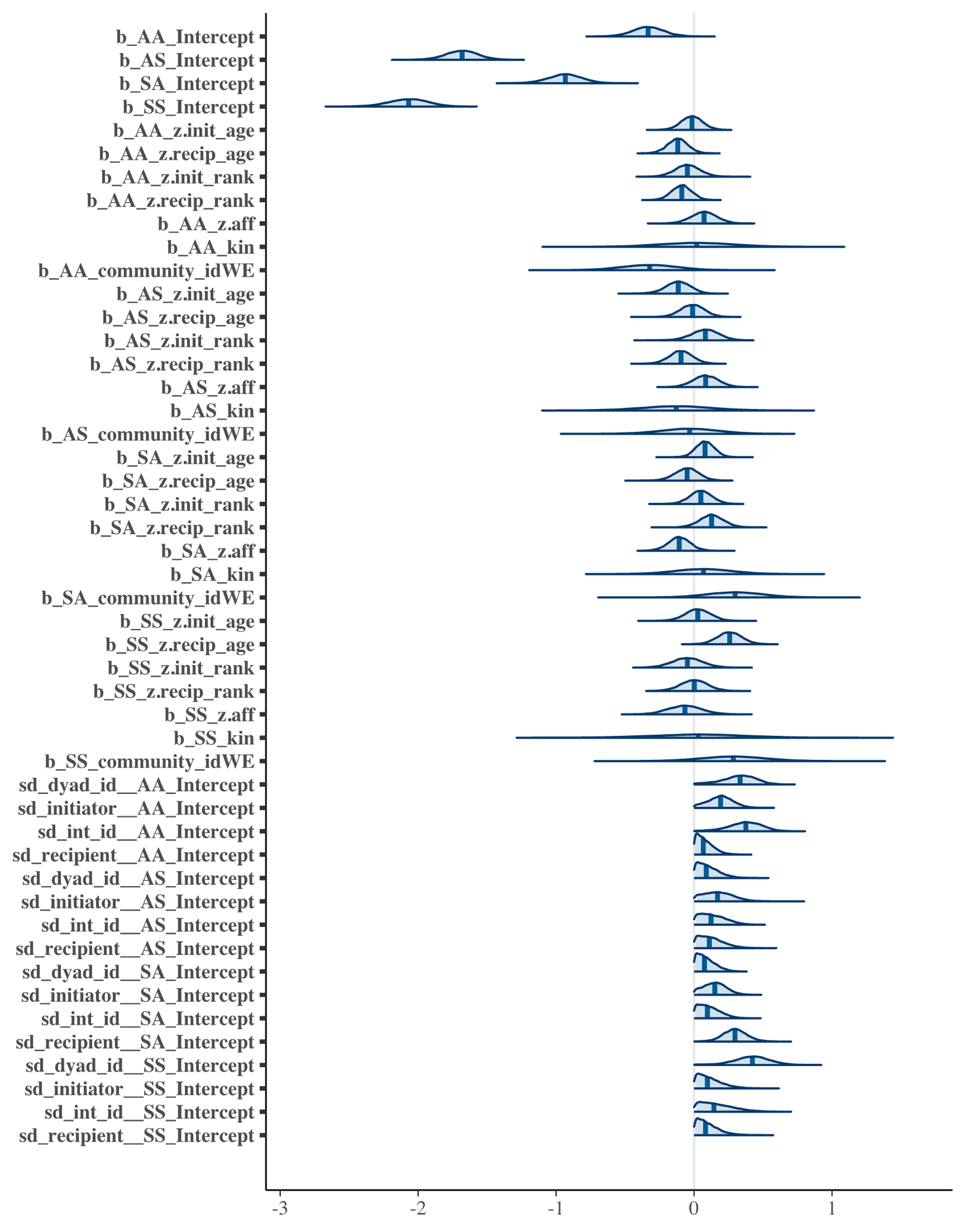
Figure S7.** Density plot of posterior distributions and 89% Credible Intervals for Bayesian generalized linear mixed model 3.

**
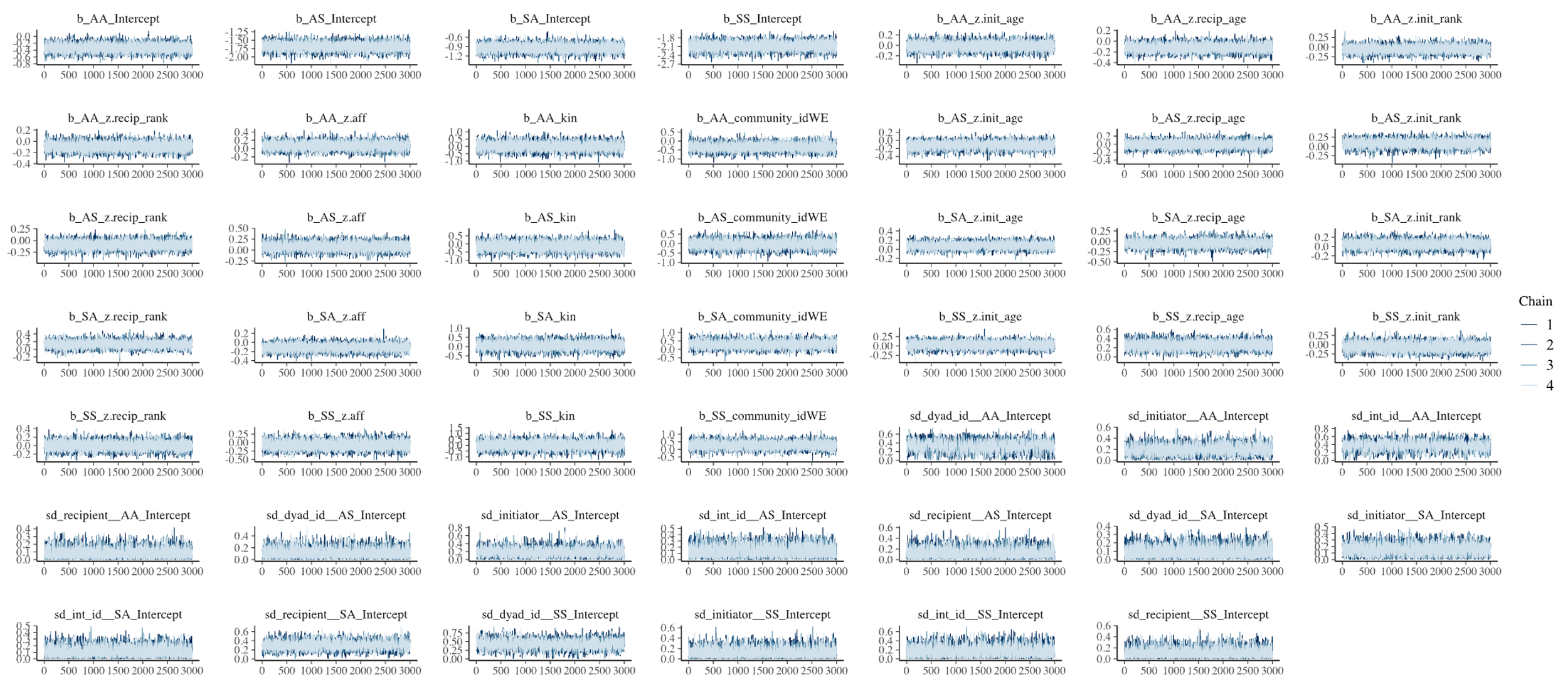
Figure S8.** MCMC trace-plots of posterior distributions of the Bayesian generalized linear mixed model 3.

**Table S6**. Temporal relationship (Model 4) testing the effect of demographic and social factors on the offset – onset timings (N = 2275). Posterior estimates of the effect of demographic and social factors on the temporal relationships.

|  | **Median estimate** | **MAD** | **89% Crl**  **[lower, upper]** | **PD** |
| --- | --- | --- | --- | --- |
| Intercept | 4.62 | 0.01 | 4.61 | 4.63 |
| Type_AA | 0.02 | 0.01 | 0.01 | 0.03 |
| Type_AS | 0.00 | 0.01 | -0.02 | 0.01 |
| Type_SA | -0.01 | 0.01 | -0.02 | 0.00 |
| Initiator age | 0.00 | 0.00 | 0.00 | 0.01 |
| Recipient age | 0.00 | 0.00 | -0.01 | 0.00 |
| Initiator rank | 0.00 | 0.01 | 0.00 | 0.01 |
| Recipient rank | 0.00 | 0.00 | -0.01 | 0.00 |
| Social bonds | 0.00 | 0.01 | 0.00 | 0.01 |
| Relatedness | 0.00 | 0.01 | -0.01 | 0.02 |
| Community ID[WE] | 0.00 | 0.01 | -0.02 | 0.01 |
| AA:Initiator age | 0.01 | 0.01 | 0.00 | 0.02 |
| AA:Recipient age | -0.01 | 0.01 | -0.02 | 0.00 |
| AA:Initiator rank | 0.00 | 0.01 | 0.00 | 0.01 |
| AA:Recipient rank | -0.01 | 0.01 | -0.01 | 0.00 |
| AA:Social bonds | 0.00 | 0.01 | -0.01 | 0.01 |
| AA:Relatedness | 0.00 | 0.02 | -0.02 | 0.02 |
| AA:Community ID[WE] | -0.02 | 0.01 | -0.03 | 0.01 |
| AS:Initiator age | 0.00 | 0.01 | -0.01 | 0.01 |
| AS:Recipient age | 0.00 | 0.01 | -0.01 | 0.01 |
| AS:Initiator rank | 0.00 | 0.01 | -0.01 | 0.01 |
| AS:Recipient rank | 0.00 | 0.01 | -0.01 | 0.01 |
| AS:Social bonds | 0.00 | 0.01 | -0.02 | 0.01 |
| AS:Relatedness | 0.01 | 0.02 | -0.02 | 0.04 |
| AS:Community ID[WE] | 0.01 | 0.02 | -0.01 | 0.03 |
| SA:Initiator age | 0.00 | 0.01 | -0.01 | 0.01 |
| SA:Recipient age | 0.00 | 0.01 | -0.01 | 0.01 |
| SA:Initiator rank | 0.00 | 0.01 | -0.01 | 0.00 |
| SA:Recipient rank | 0.00 | 0.01 | 0.00 | 0.01 |
| SA:Social bonds | 0.00 | 0.01 | -0.01 | 0.01 |
| SA:Relatedness | 0.00 | 0.02 | -0.03 | 0.02 |
| SA:Community ID[WE] | 0.00 | 0.01 | -0.01 | 0.02 |

**
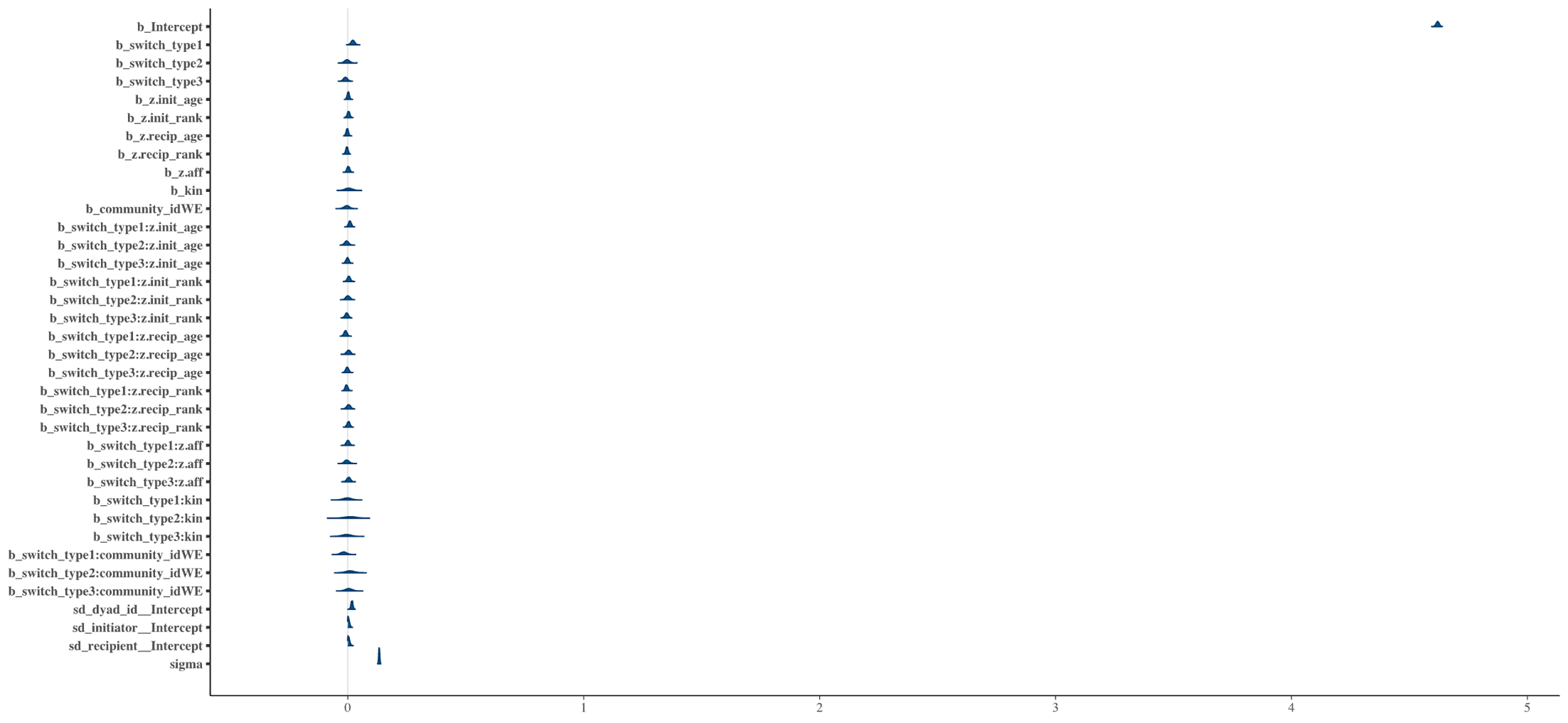
Figure S9.** Density plot of posterior distributions and 89% Credible Intervals for Bayesian generalized linear mixed model 4.

**
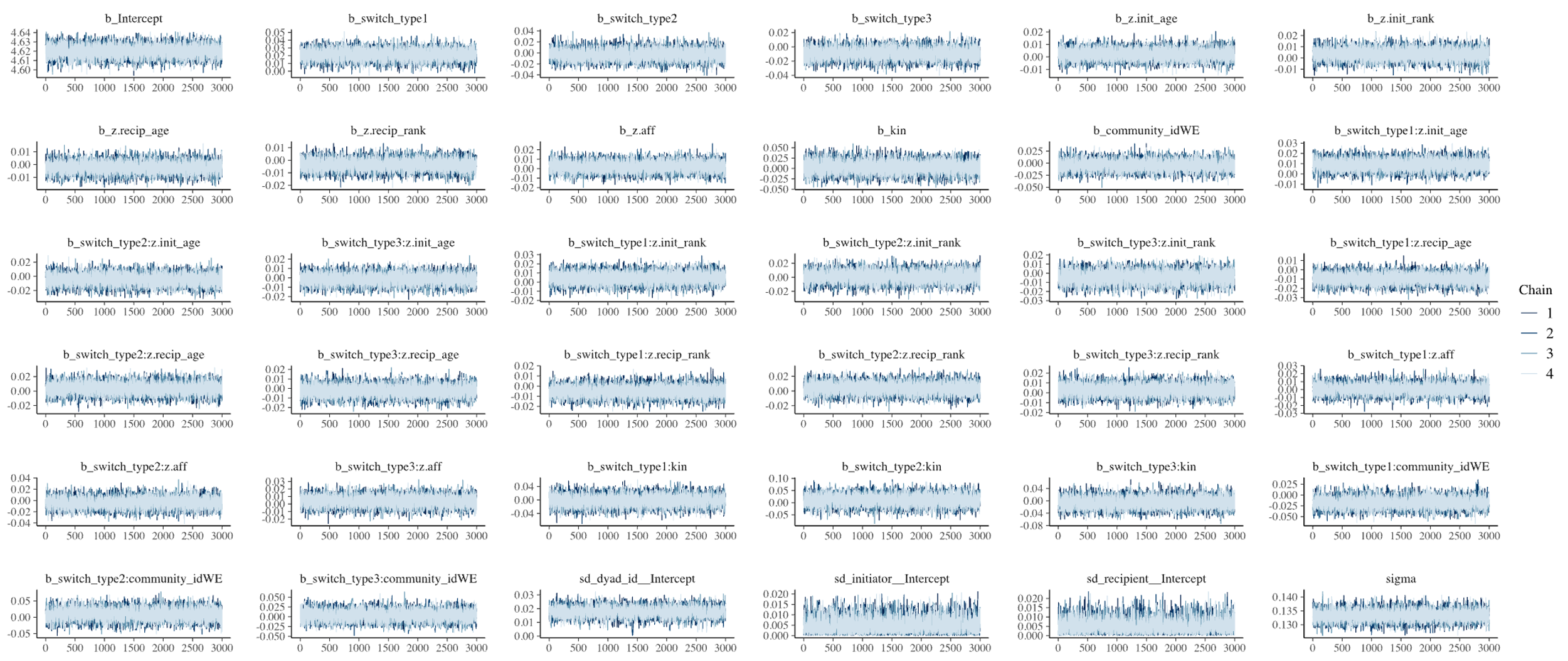
Figure S10.** MCMC trace-plots of posterior distributions of the Bayesian generalized linear mixed model 4.

1. “init_age” refers to the initiator’s age, “recip_age” refers to the recipient’s age, “init_rank” refers to the initiator’s rank, and “recip_rank” refers to the recipient’s rank. “aff” denotes social bonds, “kin” represents relatedness, and “community_id” indicates community identity. “initiator” and “recipient” refer to the identities of the initiator and recipient, respectively, while “dyad_id” represents the dyad’s identity, and “int_id” refers to interaction identity. [↑](#footnote-ref-1)
2. Not included in every model [↑](#footnote-ref-2)
